# How Patient-Specific Guides Can Ease the Learning Curve of Cortical Bone Trajectory: A Retrospective Study

**DOI:** 10.7759/cureus.109357

**Published:** 2026-05-21

**Authors:** Mark Ehioghae, Kevin Yoon, Lancelot A Benn, Jonathan P Japa, Muhamed Farhan-Alanie, Justin Hyde, Sean Bae, Ala Alshomali, Addisu Mesfin, Oliver Tannous

**Affiliations:** 1 Department of Orthopedics, MedStar Orthopedic Institute, Washington, USA; 2 Department of Orthopedics, MedStar Hospital Research Institute, Washington, USA; 3 Department of Orthopedic Spine Surgery, MedStar Hospital Research Institute, Washington, USA; 4 Department of Trauma and Orthopedics, University of Warwick, Coventry, GBR; 5 Department of Orthopedics, Georgetown University School of Medicine, Washington, USA

**Keywords:** 3d-printed surgical guides, cortical bone trajectory, learning curve, lumbar fusion, patient-specific instrumentation

## Abstract

Background: The objective of the study was to compare operative time, estimated blood loss (EBL), length of stay (LOS), and perioperative complications between lumbar fusions performed with cortical bone trajectory (CBT) screw fixation using patient-specific instrumentation (PSI) versus freehand. Secondary objectives were to quantify the learning curve and economic impact of PSI.

Methodology: We reviewed all CBT lumbar fusions performed by a single surgeon between 2018 and 2025, comparing a freehand approach with CT-guided PSI assistance. Thin-cut CT scans were used to build individualized, 3D-printed guides. Propensity matching for age, sex, and levels fused produced balanced cohorts.

Results: In total, 247 CBT fusions were analyzed: 28 (11.3%) freehand and 219 (88.7%) PSI. After the introduction of PSI, annual CBT volume increased from roughly 7 to 55 cases. Matched comparisons showed that PSI reduced mean operative time by about 33 minutes (149.5 vs. 182.5 minutes, *P* = 0.003) and lowered EBL by roughly 75 mL (*P *= 0.026). Reoperation (4 vs. 10) and wound complication (0 vs. 3) rates were lower but not statistically significant. Among two-stage procedures, infection and reoperation rates dropped from 35.7% to 7.5% (*P *< 0.001). Overall efficiency per fused level improved by 18%-25 % (*P* = 0.02).

Conclusions: Use of PSI in CBT lumbar fusion shortened operations, reduced blood loss, and maintained safety, while allowing the surgeon to perform more cases with greater consistency. The results suggest PSI smooths the CBT learning curve and may offer practical cost savings through improved operating-room efficiency.

## Introduction

Cortical bone trajectory (CBT) is an innovative screw placement technique designed to enhance fixation in lumbar spine surgery by engaging denser cortical bone. First described by Santoni et al. in 2009, CBT optimizes bone to cortical contact and minimizes soft tissue disruption by using a caudocephalad and medial-to-lateral approach [1,2]. The technique also utilizes a smaller, shorter screw to maximize thread contact with the high-density bone surface [3-5]. In combination, CBT offers enhanced fixation stability while decreasing the risk of neurovascular damage or vertebral perforation found with the traditional pedicle screw approach [4]. Past literature reviewing the use of CBT in degenerative lumbar conditions has shown equivalent, and sometimes improved, intra-operative and perioperative outcomes compared with the traditional pedicle screw approach [6-8]. However, studies were primarily performed in East-Asian cohorts by surgeons adept in the technique [9]. 

Although early evidence has shown promising results, there is a paucity of data reviewing long-term post-operative outcomes, hindering wider adoption [9-10]. Furthermore, despite the known biomechanical advantages, the unknown learning curve of the technique creates hesitancy, especially for surgeons unfamiliar with the technique. Navigation and patient-specific instrumentation (PSI) systems have emerged as potential solutions to mitigate these challenges by improving accuracy and reducing variability. In turn, PSI can ease the transition between traditional screw placement and CBT for physicians unfamiliar with the technique [11-13]. 

Thus, the primary aim of this study was to evaluate the impact of adopting the MySpine PSI system (Medacta, Frauenfeld, Switzerland) on operative efficiency and outcomes in CBT screw placement, particularly in the context of a single surgeon initially transitioning to CBT using a freehand technique. Outcomes of interest to answer this question included estimated blood loss (EBL), complications, and operative time. By comparing the freehand era with the PSI era, we seek to answer our second objective, which was to determine whether PSI can indeed alleviate the learning curve and deliver improved outcomes comparable to surgeons well versed in CBT.

## Materials and methods

Study design and setting

This was a retrospective, case-controlled study conducted by a single surgeon. The cohort was found continuously. The goal of the study was to compare two techniques of CBT screw insertion in lumbar fusion surgery. We compared CT-guided PSI using the Medacta MySpine system (Medacta) to a freehand CBT technique in lumbar fusion. Data were collected from procedures performed between May 2018 and January 2025. Institutional Review Board (IRB) approval was obtained from our institution’s review board.

Patient selection

Patients were initially stratified using respective current procedural terminology (CPT) codes for all approaches of lumbar fusion (22612/22614, 22630, 22632, 22558/22585/22586, 22633/22634). Patients were included if they underwent lumbar fusion with CBT screws by a single surgeon at the institution. Two cohorts were defined: patients receiving CT-guided MySpine PSI (further divided into single-stage and two-stage procedures) and those undergoing freehand CBT. Exclusion criteria included patients younger than 18 years, those with prior lumbar surgery, those with a history of lumbar spine trauma, and those with a preoperative primary or metastatic spinal neoplasm.

Surgical technique

In the CT-guided cohort, all patients underwent high-resolution lumbosacral CT imaging with slice thickness <1 mm to facilitate detailed three-dimensional reconstruction and preoperative planning. CT data were uploaded to the Medacta MySpine platform (Medacta) through the MySolutions interface [14-16]. Using the MySpine navigation software, the surgeon reviewed each dataset in axial, sagittal, and coronal planes to define the optimal CBT for each pedicle. Screw length and diameter were maximized while ensuring containment within the cortical corridor. Pathologic changes and anatomical variants were assessed to refine the trajectory and minimize the risk of cortical breach or neurovascular compromise.

**Figure 1:**
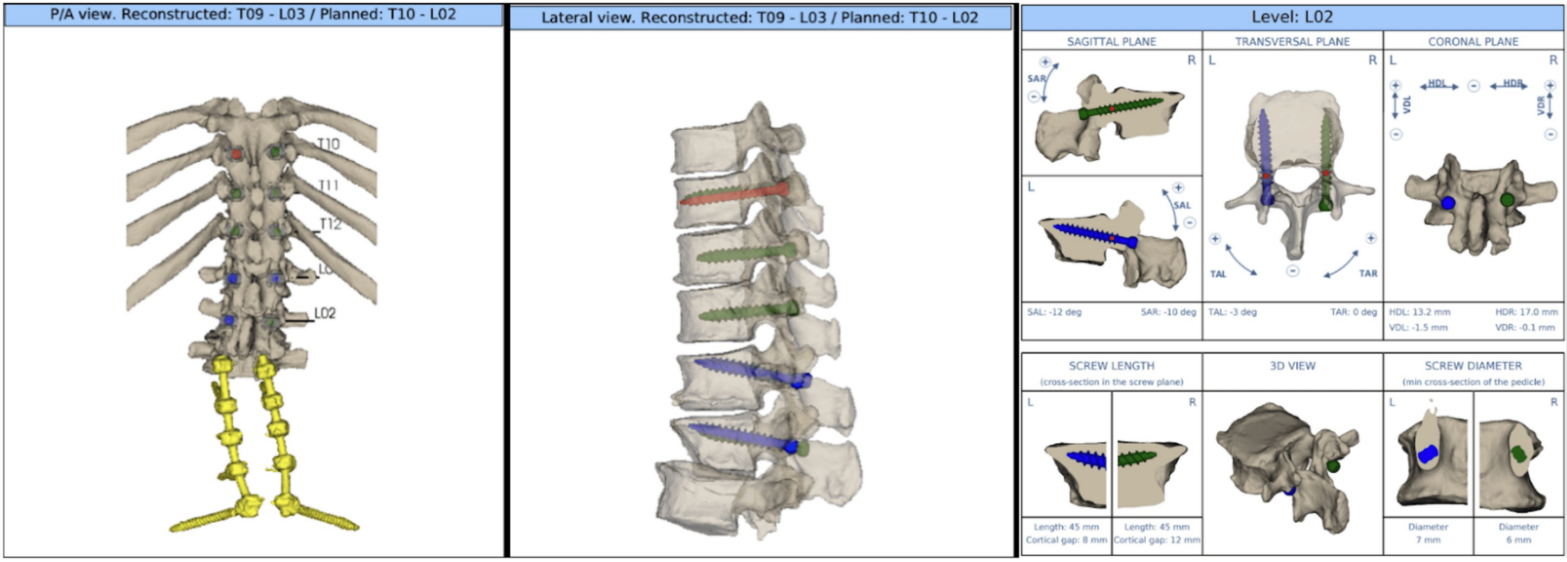
Computed tomography (CT)-guided imaging using the Medacta MySpine patient-specific instrumentation (PSI) system (Medacta, Frauenfeld, Switzerland). The system provides a visual rendering of the cortical bone trajectory (CBT) into the vertebral body in the axial, sagittal, and coronal planes before printing.

After digital planning, patient-specific navigation guides were 3D printed in Europe based on the final approved plan and delivered sterile for intraoperative use, typically within two to three weeks (Figure 1). Intraoperatively, a small (approximately 2-3 cm) midline incision was made along the spinous process, and subperiosteal dissection was performed to expose the posterior bony elements. Compared to traditional transpedicular exposure, a greater degree of soft tissue clearance over the lamina was necessary to ensure stable docking of the navigation templates.

**Figure 2:**
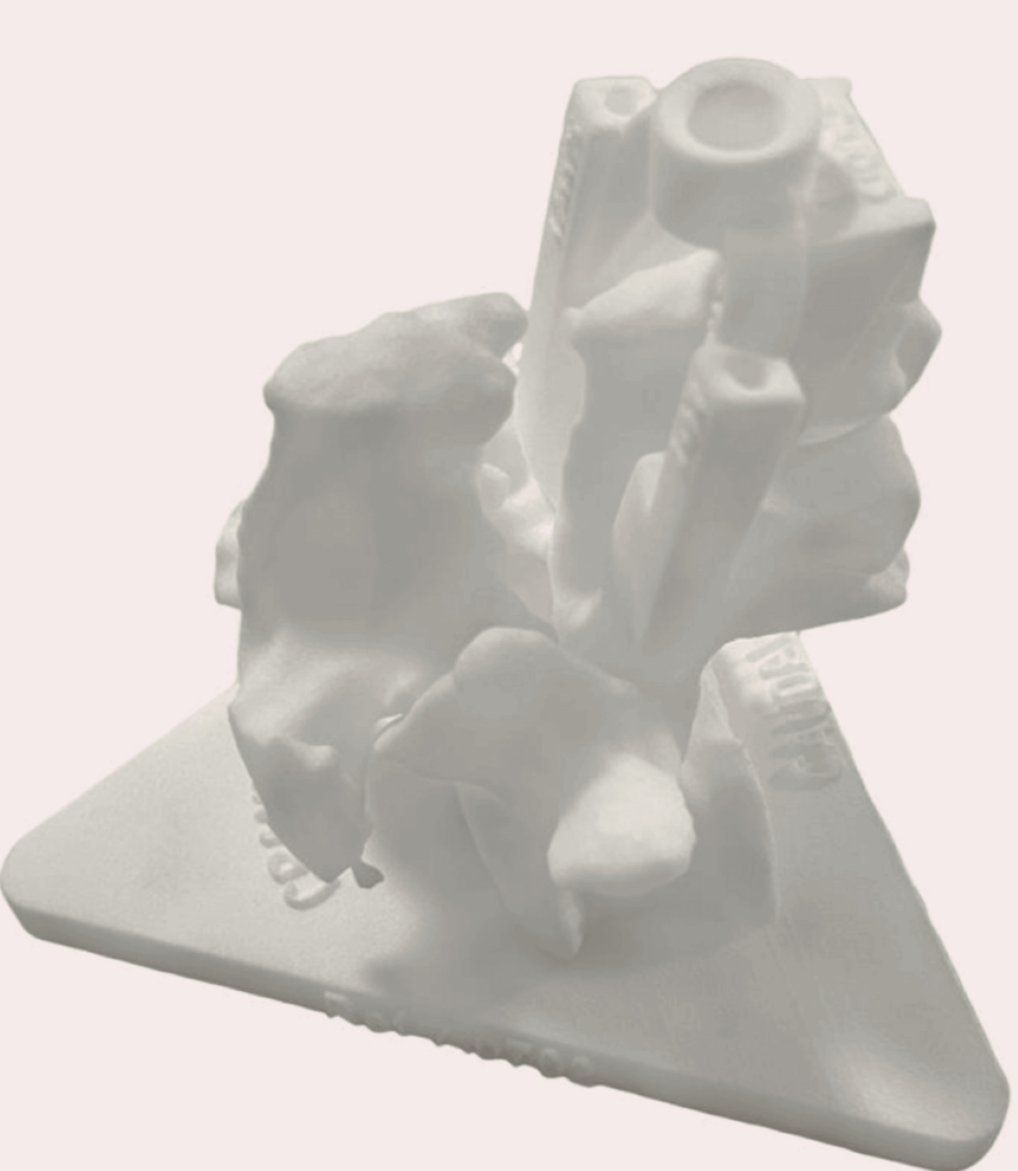
Three-dimensional (3D)-printed guide for the L2 spine.

The 3D-printed guides were positioned and secured to the posterior elements using distinct bony landmarks: spinous process, lamina, caudal laminar edge, and pars interarticularis. Surrounding osteophytes were resected as needed to allow for the precise seating of the guide. Once a proper fit was confirmed visually and with lateral fluoroscopy, drilling was performed through the guide sleeves in standard fashion. Each pedicle was drilled and tapped sequentially; tracts were palpated with a ball-tipped probe to confirm cortical integrity before screw insertion. Preplanned CBT screws were then placed bilaterally at the intended fusion levels (e.g., L1-L2), typically ranging from 6.0 × 40 to 45 mm in length (Figure 2). In the freehand group, screws were placed using a conventional CBT technique without CT-based planning or patient-specific guides, relying instead on anatomical landmarks and surgeon experience for trajectory determination. Requirement of a staged procedure was based on the surgeon's discretion, made based on the severity of clinical symptoms, imaging characteristics, patient goals postoperatively, and patient medical history and ability to tolerate multi-step procedures. 

Outcomes and variables

Primary outcomes included operative time, estimated blood loss, length of stay, intra-/peri-operative complications, and reoperation rates. Complications included infections, wound-related reoperations, intra-operative durotomy, and common medical complications, including myocardial infarction, deep vein thrombosis, and transfusions, were recorded. Fluoroscopy time was noted when documented. Patient information regarding age, gender, race, follow-up period, and levels fused was also noted.

To calculate efficiency, operative time was normalized to levels fused to identify the time per vertebra. Learning curve was identified by analyzing a rolling 10-case median operative time using five cases before and five cases at the time point, and comparing changes after initial transition to CBT and following throughout the duration of the study. 

Statistical analysis

All statistical analyses were performed using SPSS version 29 (IBM Corp., Armonk, NY). Demographic variables were first compared between single-stage PSI and free-hand patients. Continuous variables were first summarized as medians and compared using the Mann-Whitney U test. Categorical variables were compared with chi-square analysis and Fisher’s exact test for nonparametric variables. Due to the large number of comparisons, a Bonferroni correction was performed. A nearest-neighbor propensity score matching was then performed using age, gender, and levels fused using a caliper width of 0.1. Patients with missing variables for matching were removed from post-PSM analysis. A standard mean difference of <0.01 was achieved for all variables matched for. An alpha level of 0.05 was considered statistically significant for all comparisons.

Post-PSM, logistic and linear regression analyses using Firth regression and robust analysis of covariance (ANCOVA) were attempted. However, due to the limited sample size and low incidence of complications, the regression results were deemed unstable. Therefore, all outcomes were compared using the aforementioned methods.

## Results

Baseline demographics 

A total of 247 CBT screw cases were analyzed: CT-guided-with-PSI patients (single-stage, *n* = 152, 61.5%; two-stage, *n* = 67, 27.1%) and freehand patients (*n* = 28, 11.3%). The cohorts were similar in baseline demographics. The CT-guided-with-PSI group had a mean age of 64.72 ± 11.16 years and was primarily female (*n* = 149, 68.04%) and African-American patients (*n* = 123, 56.16%). Within this cohort, the most common indication was radiculopathy (*n* = 130, 59.36%), followed by stenosis (*n* = 125, 57.08%). The most common procedure was TLIF (*n* = 132, 60.27%), and a minority involved the sacrum (*n* = 70, 31.96%). Mean follow-up duration was 11 weeks (range: 4-18 weeks).

On the other hand, the freehand group had a mean age of 63 ± 11.46 years and was primarily female (*n* = 16, 57.1%) and African-American patients (*n* = 10, 35.71%). The most common indication was stenosis (*n* = 23, 82.14%), followed by radiculopathy or spondylolisthesis (*n* = 19, 67.86% each). The most common procedure was ALIF (*n* = 26, 92.86%), and nine cases involved the sacrum (*n* = 9, 32.14%). Mean follow-up duration was 38 weeks (range: 14.63-47.25 weeks). Total demographic data are shown in Table 1.

**Table 1:** Demographic univariable comparison of CT-guided versus freehand patient cohorts. CT, computed tomography

Variable	Computed tomography (CT) image-guided cases (*n* = 219)	Freehand (*n* = 28)	*χ*²(df)	*P*-value
Average age (SD)	64.72 (11.16)	63 (11.46)		0.458
Gender (*n*, %)			0.54 (1)	0.462
Male	68 (31.05%)	12 (42.86%)		
Female	149 (68.04%)	16 (57.14%)		
Race (*n*)			0.00 (1)	1
African American	123 (56.16%)	10 (35.71%)		
Non-African American	41 (18.72%)	3 (10.71%)		
Levels fused (*n*, %)			6.00 (4)	
1	121 (55.25%)	22 (78.57%)		
2	61 (27.85%)	5 (17.86%)		
3	33 (15.07%)	1 (3.57%)		
4	2 (0.91%)	0 (0.00%)		
5	1 (0.46%)	0 (0.00%)		
Lumbar	204 (93.15%)	27 (96.43%)	0.44 (1)	0.507
Sacral	70 (31.96%)	9 (32.14%)	0.00 (1)	0.989
Stenosis	125 (57.08%)	23 (82.14%)	6.17 (1)	0.013
Radiculopathy	130 (59.36%)	19 (67.86%)	0.75 (1)	0.387
Spondylolisthesis	108 (49.32%)	19 (67.86%)	3.40 (1)	0.065
Other clinical findings	20 (9.13%)	2 (7.14%)	0.00 (1)	1
Procedure			16.01 (4)	0.003
Anterior lumbar interbody fusion	2 (0.91%)	26 (92.86%)		
Circumferential lumbar fusion	4 (1.83%)	0 (0.00%)		
Posterior lumbar fusion	80 (36.53%)	1 (3.57%)		
Transverse lumbar interbody fusion	132 (60.27%)	0 (0.00%)		
Other	0 (0.00%)	1 (3.57%)		
Postop complication			20.51 (9)	
Adjacent segment disease	23 (10.50%)	5 (17.86%)		
Wound dehiscence	2 (0.91%)	1 (3.57%)		
Heart failure	0 (0.00%)	1 (3.57%)		
Radiculopathy/Myelopathy	4 (1.83%)	0 (0.00%)		
Pulmonary embolism	1 (0.46%)	0 (0.00%)		
Pain	18 (8.22%)	6 (21.43%)		
Pseudoarthrosis	5 (2.28%)	0 (0.00%)		
Tumor	5 (2.28%)	0 (0.00%)		
Other	3 (1.37%)	1 (3.57%)		
Intra-op complication			1.42 (3)	
Dural tear	11 (5.02%)	1 (3.57%)		
Deep vein thrombosis	1 (0.46%)	0 (0.00%)		
Foley problem	1 (0.46%)	1 (3.57%)		
Pedicle split	1 (0.46%)	0 (0.00%)		

Outcomes 

After propensity matching, there were 28 single-stage CT-guided (CT-single) patients and 28 freehand patients. When comparing the two groups, there was significantly lower estimated blood loss (EBL) in the CT-single group compared with the freehand group (100 mL vs. 175 mL, *P* = 0.026). There was also a significantly shorter median operative time (149.5 minutes vs. 182.5 minutes, *P* = 0.003). In addition, there was a non-significant decrease in reoperations in the CT-single group (4/28, 14.29%, vs. 10/28, 35.71%, *P* = 0.121) and in wound complications (0/28, 0.00%, vs. 3/28, 10.71%, *P* = 0.236). Follow-up duration also differed significantly between groups (11 weeks vs. 38 weeks, *P* = 0.002). Results are shown in Table 2, and changes in blood loss and operative time before and after adoption of PSI are shown in Tables 2-3.

**Table 2:** Non-propensity score-matched comparison of single-stage CT-guided versus freehand techniques. ^†^Late onset of data collection limited cases with available fluoroscopy time data. Values are primarily descriptive rather than suitable for statistical analysis. CT, computed tomography

Outcome	CT single stage (*n*)	CT single stage (median)	CT single stage (range)	Freehand (*n*)	Freehand (median)	Freehand (range)	χ² (df)	*P*-value
Age (years)	151	65	60.0-72.0	28	64.5	56.8-71.2		0.7
Instrumented levels (count)	151	1	1.0-2.0	28	1	1.0-1.0		0.279
Estimated blood loss (mL)	150	100	100-200	20	175	137.5-250		0.044
Operative time (minutes)	151	150	132-184	27	183	166-210		<0.001
Length of stay (days)	149	4	3.0-5.0	27	3	2.5-4.0		0.077
Intra-op complications	151	5.3% (8/151)	-	28	7.1% (2/28)	-	0.20 (1)	0.656
Reoperation (any during follow-up)	151	17.9% (27/151)	-	28	35.7% (10/28)	-	10.83 (1)	0.001
Fluoroscopy time (seconds)†	21	30	25.2-42.3	2	76	75-77		-
Post-operative complication	152	17.9% (27/151)	-	28	35.7% (10/28)	-	10.83 (1)	0.001
Infection/Wound Dehiscence	152	5.3% (8/152)	-	28	10.7% (3/28)	-	0.77 (1)	0.381
Follow-up (weeks)	150	11	6-23.25	28	38	14.63-47.25		<0.001

**Table 3:** Propensity-score-matched comparison of CT-guided single-stage versus freehand techniques. CT, computed tomography

Outcome	CT single stage (*n*)	CT single stage (median)	CT single stage (range)	Freehand (*n*)	Freehand (median)	Freehand (range)	χ²(df)	*P*-value
Age (years)	28	62.5	57-72	28	66	57-72.75		0.883
Instrumented levels (count)	28	1	1-1.75	28	1	1-1		0.763
Estimated blood loss (mL)	28	100	50-100	28	175	112.5-250		0.026
Operative time (minutes)	28	149.5	132.75-179.75	28	182.5	166-226.5		0.003
Length of stay (days)	28	3	2-4	27	3	2.25-4		0.931
Intra-op complications	28	7.14% (2/28)	-	28	7.14% (2/28)	-	0.00 (1)	1
Reoperation (any during follow-up)	28	14.3% (4/28)	-	28	35.7% (10/28)	-	2.40 (1)	0.121
Postoperative complication	28	14.3% (4/28)	-	28	35.7% (10/28)	-	2.40 (1)	0.121
Follow-up (weeks)	28	38	14.63-47.25	28	38	15.25-46.5		0.002
Infection/wound dehiscence	28	0% (0/28)	-	28	10.7% (3/28)	-	1.40 (1)	0.236

After propensity matching, there were 22 two-stage (CT-double) patients and 28 freehand patients. When comparing the two groups, the CT-double group had a significantly longer median operative time (281 minutes (range: 235-335 minutes) vs. 182.5 minutes (range: 166-226.5 minutes), *P* < 0.001) and significantly lower reoperation/postoperative infection rates (both 1/22, 4.5% vs. 10/28, 35.7%, *P* < 0.001). The freehand group had a significantly shorter median length of stay (3 days vs. 6 days, *P* < 0.001), a significantly longer median follow-up period (39.5 weeks vs. 11.5 weeks, *P* < 0.001), and a nonsignificantly higher infection rate (0/22, 0.0%, vs. 3/28, 10.71%, *P* = 0.246). Full results are shown in Tables 4-5.

**Table 4:** Non-propensity score-matched comparison between two-stage CT-guided versus freehand techniques. CT, computed tomography

Outcome	CT two-stage (*n*)	CT two-stage (median)	CT two-stage (range)	Freehand (*n*)	Freehand (median)	Freehand (range)	χ²(df)	*P*-value
Age (years)	67	69	60-74	28	66	57-72.75		0.211
Instrumented levels (count)	67	2	2-3	28	1	1-1		<0.001
Estimated blood loss (mL)	67	230	150-330	28	175	112.5-250		0.157
Operative time (minutes)	64	281	235-335	28	182.5	166-226.5		<0.001
Length of stay (days)	67	6	5-8	28	3	2.25-4		<0.001
Intra-op complications	67	9.0% (6/67)	-	28	7.14% (2/28)	-	0.00 (1)	1
Reoperation (any during follow-up)	67	7.5% (5/67)	-	28	35.7% (10/28)	-	10.83 (1)	0.001
Postoperative complication	67	7.5% (5/67)	-	28	35.7% (10/28)	-	10.83 (1)	0.001
Follow-up (weeks)	63	11	4-18	28	38	14.63-47.25		<0.001
Infection/wound dehiscence	67	0% (0/67)	-	28	10.7% (3/28)	-	5.09 (1)	0.024

**Table 5:** Propensity-score-matched comparison of two-stage CT-guided versus freehand techniques. CT, computed tomography

Outcome	CT two-stage (*n*)	CT two-stage (median)	CT two-stage (range)	Freehand (*n*)	Freehand (median)	Freehand (range)	χ²(df)	*P*-value
Age (years)	22	60	51.75-71.75	28	64.5	56.25-71.75		0.519
Instrumented levels (count)	22	1	1-2	28	1	1-1		0.582
Estimated blood loss (mL)	22	150	80-312.5	28	175	112.5-250		0.82
Operative time (minutes)	22	266	194.25-326.5	28	183	166-214		0.002
Length of stay (days)	22	5	3.75-8.0	28	3	2-4		<0.001
Intra-op complications	22	4.5% (1/22)	-	28	7.14% (2/28)	-	0.00 (1)	1
Reoperation (any during follow-up)	22	4.5% (1/22)	-	28	35.7% (10/28)	-	6.04 (1)	0.014
Postoperative complication	22	4.5% (1/22)	-	28	35.7% (10/28)	-	6.04 (1)	0.014
Follow-up (weeks)	22	11.5	3.75-18	28	39.5	16.5-48.5		<0.001
Infection/Wound Dehiscence	22	0% (0/22)	—	28	10.7% (3/28)	—	1.35 (1)	0.246

Pre-propensity score-matched single- versus two-stage groups were compared. The two-stage group showed significantly higher numbers of levels instrumented, EBL, operative time, and length of stay. Results of the comparison are shown in Table 6.

**Table 6:** Non-propensity score-matched operative outcomes between single- versus two-stage CT-guided procedures. CT, computed tomography

Outcome	CT single stage (*n*)	CT single stage (median)	CT single stage (range)	CT two-stage (*n*)	CT two-stage (median)	CT two-stage (range)	χ²(df)	*P*-value
Age (years)	151	65	60.0-72.0	66	69	57-75		0.159
Instrumented levels (count)	151	1	1.0-2.0	66	2	1-3		<0.001
Estimated blood loss (mL)	150	100	100-200	66	217	145-330		<0.001
Operative time (min)	151	150	132-184	63	278	235-334		<0.001
Length of stay (days)	149	4	3.0-5.0	66	6	4-8		<0.001
Intra-op complications	151	5.3% (8/152)	-	66	10.0% (6/66)	-	0.81 (1)	0.37
Reoperation (any during follow-up)	151	17.9% (27/151)	-	66	7.6% (5/66)	-	3.51 (1)	0.061
Follow-up (weeks)	150	11	6-23.25	65	11	4-17.5		0.383
Postoperative complication	149	17.9% (27/151)	-	66	7.6% (5/66)	-	4.22 (1)	0.04
Infection/Wound dehiscence	152	5.3% (8/152)	-	66	0% (0/66)	-	2.57 (1)	0.109

Efficiency

Due to the inherent difficulties in comparing two-stage groups versus a single-stage freehand group, an efficiency-per-unit-time analysis was performed by comparing operative time based on levels fused and blood loss. Analysis showed a significantly lower operative time per level fused (*P* = 0.020). Results are shown in Table 7.

**Table 7:** Two-stage versus freehand operative efficiency normalized to vertebral levels. CT, computed tomography

Metric	CT two-stage (*n*)	CT two-stage (Median)	CT two-stage (Range)	Freehand (*n*)	Freehand (Median)	Freehand (Range)	*P*-value
Operative time per level (minutes/level)	64	126.0	104.7-168.1	27	150.0	100-200	0.020
Estimated blood loss per level (mL/level)	67	102.5	73.3-150.0	20	166.0	25-250	0.087
Levels per hour	64	0.48	0.36-0.56	27	0.36	0.31-0.43	0.0256

## Discussion

**Figure 3:**
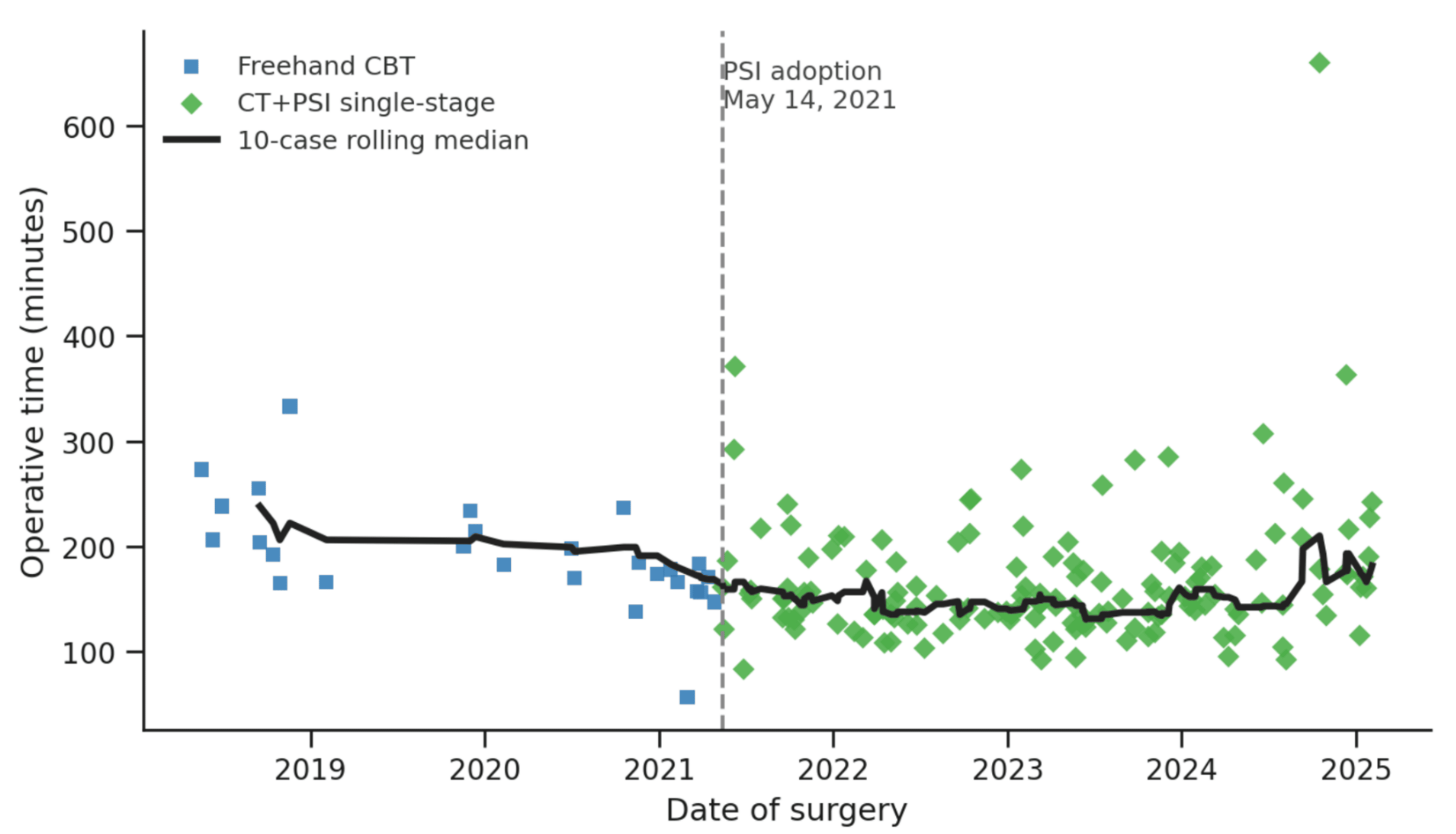
Operative time over time with the PSI adoption threshold (single surgeon). Scatter points represent individual operative times for freehand (pre-PSI) and CT+PSI single-stage (post-PSI) cases. The solid line represents the 10-case rolling median. The dashed vertical line indicates PSI adoption (May 14, 2021). PSI, patient-specific instrumentation; CBT, cortical bone trajectory

To our knowledge, this is one of the first studies to review the impact of patient-specific imaging on the learning curve associated with the CBT technique. The analysis over the surgeon’s timeline demonstrated that the introduction of PSI was associated not only with an immediate stepwise reduction in operative time but also with a flattening of the learning curve (Figure 3). The same findings were seen even in larger, two-stage procedures, where our findings showed improved efficiency when normalized to levels fused, highlighting the benefits of PSI in larger, more complex, multi-level procedures. Notably, the adoption of PSI corresponded with a substantial increase in CBT utilization, from an average of seven cases per year during the freehand era to 55 cases per year after PSI implementation. This sharp rise likely reflects a tangible increase in surgeon confidence and efficiency supported by enhanced safety margins, reproducibility, and consistency of screw placement afforded by PSI-guided planning. Prior studies have shown that proficiency in freehand CBT can require dozens of cases, with early attempts carrying higher risks of cortical breach and neurovascular compromise [7,17-18]. By contrast, PSI appears to accelerate the acquisition of technical proficiency, allowing surgeons - even those relatively early in practice - to adopt CBT more readily and perform it more consistently. Similar findings were seen with estimated blood loss, although less pronounced. Variability in EBL may correlate to inherent patient differences - especially in an American cohort where risk factors such as obesity and diabetes mellitus can significantly increase risk of intra-operative bleeding. 

Importantly, the transition to PSI did not lead to an increase in complication rates, even though our cohort represented a demographically and clinically distinct U.S. population compared with prior studies conducted primarily in Asian and European settings [19,20]. This aligns with prior studies in which Black/African-American patients did not demonstrate differences in outcomes after lumbar fusion [21,22]. Our observed infection and reoperation rates were in line with or slightly better than those reported in the broader literature, reinforcing the notion that PSI not only improves efficiency but does so without compromising safety [8,23]. Notably, lower complications remained constant for both one- and two-stage procedures compared with the freehand technique, even with a shorter median follow-up time for the freehand PSM group. Lower complication rates should be expected with the combination of PSI and 3D-printed guides. Preoperative imaging studies not only guide the trajectory but also bring to attention points of potential complication, such as bone defects or areas of low bone mineral density. 3D-printed guides then help execute screw placement with decreased variability versus freehand placement. Our study also found that long-term outcomes differed from prior findings where CBT had worsened pain scores, higher rates of adjacent segment disease requiring operative treatment, but no significant differences in pseudoarthrosis and adverse events compared with the traditional pedicle screw approach [8,24,25]. The underlying factors driving these long-term outcomes remain unclear. The relative novelty of the CBT approach may contribute to this finding, as even experienced surgeons are likely still progressing through the later stages of the learning curve amid the rapid expansion of its adoption.

It is worth emphasizing the economic implications of introducing PSI in CBT-based lumbar fusion. A systematic review of surgical cost-analysis methods emphasized that operative time remains one of the most significant cost drivers in surgical care [26]. From a hospital and surgeon perspective, a consistent reduction of approximately 33 minutes per case translates into substantial cost savings, given that typical operating room (OR) expenses in U.S. acute-care hospitals range from $37 to $46 per minute [27, 28]. Based on this estimate, each PSI-guided procedure could reduce direct operative costs by $1,221 to $1,518 per case. Across the 219 PSI-assisted cases included in this study, this equates to an overall potential savings of $267,000 to $332,000 in OR time alone. Over multiple cases, such time efficiency may also permit an additional lumbar fusion for every four to five PSI-guided cases compared with freehand procedures. From the patient and health-system standpoint, the combination of shorter operative duration, lower blood loss, fewer complications, and reduced hospital length of stay further lowers total episode-of-care expenditures and aligns with current value-based reimbursement models.

Although studies specifically evaluating the learning curve are limited, another potential surrogate may be found in changes in screw accuracy. For instance, prior cadaveric studies, such as those performed by Cool et al., found that the use of similar 3D-printed guides for fixation in the thoracolumbar region resulted in almost 100% placement within the *safe region* of the vertebrae [29,30]. Similar studies by Fujita et al. and Matsukawa et al. echoed these findings in non-cadaveric lumbar fusion, demonstrating significantly improved screw accuracy compared with freehand techniques (*P* < 0.001), although these studies were performed by experienced surgeons [19,20]. As technological advancements continue, one avenue for future research could involve evaluating the integration of 3D modeling with virtual reality tools to develop new methods for improving procedural learning through enhanced visual and tactile precision.

Limitations and future directions

We acknowledge that our study is limited by its retrospective design and single-surgeon setting, which may affect generalizability and are major sources of bias. Due to the single-surgeon nature and lack of surgical standardization, we must reiterate that our findings should not be overinterpreted, especially regarding the learning curve and efficiency effects. Learning changes with additional surgeons would further validate the findings of the current study. Differences in follow-up period between PSI and non-PSI cohorts also introduce selection bias within the available cohorts. Additionally, due to the small number of events, it was not possible to analyze individual complications. The low number of PSI patients available also resulted in an often unstable statistical model when attempting multivariable analyses. 

Additionally, while we observed clear operative time improvements and a stabilized learning curve, we did not have data on accuracy metrics such as breach rates, which have been highlighted in other PSI studies as an area of potential benefit. Our study was also unable to accurately depict radiation risk, a known associated sequela of CT-guided PSI. Lastly, one major limitation of PSI utilization is the upkeep required for CT-based guides [15]. Thus, future studies reviewing the cost of procedures with and without 3D-printed guides, as well as the additional facility costs of CT imaging, should be considered to illustrate obstacles to large-scale adoption.

## Conclusions

In conclusion, our findings contribute to the growing body of evidence supporting the use of PSI in CBT screw placement, demonstrating that it can reduce operative times and complications in lumbar fusion, indicating a flattening of the learning curve while maintaining safety standards. However, the generalizability of our findings is limited by the single-surgeon and retrospective nature of our study. Future prospective studies and multi-surgeon analyses are needed to confirm these benefits across different settings and improve the generalizability of our findings.

Our study provides a small glimpse into the experience of physicians transitioning from traditional pedicle screw placement, and our findings indicate the potential benefit of PSI for CBT in maintaining safety standards during the initial learning period.
